# Culture-independent method for identification of microbial enzyme-encoding genes by activity-based single-cell sequencing using a water-in-oil microdroplet platform

**DOI:** 10.1038/srep22259

**Published:** 2016-02-26

**Authors:** Kazuki Nakamura, Ryo Iizuka, Shinro Nishi, Takao Yoshida, Yuji Hatada, Yoshihiro Takaki, Ayaka Iguchi, Dong Hyun Yoon, Tetsushi Sekiguchi, Shuichi Shoji, Takashi Funatsu

**Affiliations:** 1 Graduate School of Pharmaceutical Sciences, The University of Tokyo, 7-3-1, Hongo, Bunkyo-ku, Tokyo 113-0033, Japan; 2 Japan Agency for Marine-Earth Science and Technology, 2-15 Natsushima-cho, Yokosuka-shi, Kanagawa 237-0061, Japan; 3 Department of NanoscieWnce and Nanoengineering (ASE Graduate School), Waseda University, 3-4-1 Okubo, Shinju-ku, Tokyo 169-8555, Japan; 4 Research Organization for Nano & Life Innovation, Waseda University, 513, Waseda-tsurumaki-cho, Shinjuku-ku, Tokyo, 162-0041, Japan

## Abstract

Environmental microbes are a great source of industrially valuable enzymes with potent and unique catalytic activities. Unfortunately, the majority of microbes remain unculturable and thus are not accessible by culture-based methods. Recently, culture-independent metagenomic approaches have been successfully applied, opening access to untapped genetic resources. Here we present a methodological approach for the identification of genes that encode metabolically active enzymes in environmental microbes in a culture-independent manner. Our method is based on activity-based single-cell sequencing, which focuses on microbial cells showing specific enzymatic activities. First, at the single-cell level, environmental microbes were encapsulated in water-in-oil microdroplets with a fluorogenic substrate for the target enzyme to screen for microdroplets that contain microbially active cells. Second, the microbial cells were recovered and subjected to whole genome amplification. Finally, the amplified genomes were sequenced to identify the genes encoding target enzymes. Employing this method, we successfully identified 14 novel β-glucosidase genes from uncultured bacterial cells in marine samples. Our method contributes to the screening and identification of genes encoding industrially valuable enzymes.

Enzymes have been used increasingly in a wide range of industrial applications because of their prominent properties, such as substrate specificity and high activity, under mild pH values, temperatures and pressures. Most enzymes are derived from environmental microbes^1^, and industrially valuable enzymes are often identified in novel microbes. However, such resources cannot be completely accessed by culture-based methods because the majority of microbes are unculturable in the laboratory.

To overcome this limitation, metagenomics, which involves culture-independent genomic analysis of the microbial community^2^, has been applied to access vast untapped genetic resources^3^. In metagenomic approaches, two strategies are generally used to screen and identify novel genes from environmental DNA libraries: sequence-based and activity-based screening^4^,^5^. Sequence-based screening has led to the effective identification of enzyme-encoding genes based on sequence homology. In particular, recent developments in high-throughput sequencing technologies have considerably advanced sequence-based screening^6^. Nevertheless, operating costs for sequencing to obtain large amounts of sequencing data are still high, and sequence assembly requires lengthy computation time. In contrast, activity-based screening requires the cloning of environmental DNA fragments into vectors to screen the clones expressing selected enzymatic activity. However, it is well known that this approach is hampered by problems with gene expression in heterologous hosts^5^. In addition, a small number of clones expressing selected enzymatic activity have to be selected from several colonies (e.g. 4 hits from 389,000 clones)^7^. In both sequence- and activity-based screening, it is technically difficult to obtain enzyme-encoding genes from rare environmental microbial cells because the genes present are roughly proportionate to the population frequency of each microbe.

Single-cell genomics is a powerful emerging technique, which permits culture-independent characterisation of uncultured microbial cells^8^,^9^,^10^,^11^,^12^,^13^. It involves single cell isolation, followed by whole genome amplification and sequencing. In combination with single-cell-based screening for enzymatic activity, a single-cell genomic strategy will be a promising approach to identify novel enzyme-encoding genes from environmental microbes, including rare and uncultured microbial cells, without prior cultivation. Fluorescence-activated cell sorting (FACS) has been the most commonly used high-throughput approach to separate individual bacterial cells^9^,^10^,^11^,^12^,^13^. However, FACS has several technical problems for the sorting of microbial cells. Due to the lack of visual confirmation of cell identity, non-cellular fluorescent particles present in environmental samples can be sorted along with targeted microbial cells^14^,^15^. In addition, FACS retains a low efficiency in recovering rare cells because it requires visual inspection and manual gating of one- or two-dimensional projections of the data to identify the cell subsets of interest^16^,^17^.

Here we present a methodological approach for the identification of genes that encode metabolically active enzymes in environmental microbes by a combination of activity-based single-cell screening using microdroplets and single-cell genome sequencing (Fig. 1a). Despite being based on a combination of formerly established procedures, this method can be considered as an extended version of *in vitro* compartmentalization, which uses compartmentalization to link genotype and phenotype^18^,^19^,^20^. As a proof-of-concept experiment, we applied our method to obtain novel β-glucosidase (BGL) genes from bacteria in seawater samples. We identified 14 novel BGL genes from uncultured marine bacterial cells.

## Methods

### Preparation of environmental samples

Surface seawater was collected from the coast of Tokyo Bay, Japan (35° 19.170′ N, 139° 39.068′ E) in March 2014. The surface seawater was passed through a 41-μm nylon net filter (Merck Millipore) to separate large particles and debris. The aliquot (approximately 100 mL) was concentrated to approximately 9 mL (approximately 11-fold) by centrifugal ultrafiltration using a 10-kDa pore membrane (Amicon Ultra-15, Merck Millipore). Deep seawater was collected at a depth of 857 m off Hatsushima Island, Sagami Bay, Japan (35° 0.948′ N, 139° 13.310′ E) in April 2014. The deep seawater was passed through a 20-μm nylon net filter (Merck Millipore) and a 10-μm Omnipore membrane filter (Merck Millipore). The aliquot (approximately 200 mL) was concentrated to approximately 0.5 mL (approximately 400-fold) by centrifugal ultrafiltration. Ultrafiltration was performed at 5,000 *g* for 1–2 h at 4 °C.

### Generation of water-in-oil microdroplets

Water-in-oil (W/O) microdroplets were generated using a microfluidic device with a flow-focusing geometry, which consists of two channels intersecting in a cross^21^ (Supplementary Fig. S1). The width of the main channels was 100 μm, and the width at the flow-focusing constrictions was 40 μm. The height of all channels was 50μm. The device was built from polydimethylsiloxane (PDMS) using standard soft-lithography and mould-replica techniques, as described elsewhere^22^. In brief, PDMS base and a curing agent (SILPOT 184 W/C, Dow Corning Toray) were mixed at a 10:1 (w/w) ratio, degassed, poured over the mastermould and baked at 110 °C for 1 h. After sealing the PDMS device with a coverslip, the channel surface was treated with a solution of 0.1% (heptadecafluoro-1,1,2,2-tetrahydrodecyl)dimethylchlorosilane (Gelest) in ethanol, followed by washing with ethanol. The microfluidic device was then baked at 80 °C for 1 h. This treatment was required for the preferential wetting of the oil solution (see below) on the channel walls to generate stable W/O microdroplets^23^.

The aqueous solution was composed of concentrated seawater sample (approximately 1 × 10^7^ cells/mL) containing 2 mM fluorescein di-β-D-glucopyranoside (FDGlu; Marker Gene Technologies), whereas the oil solution consisted of mineral oil (Sigma-Aldrich) containing 4% (v/v) ABIL EM90 (Evonik Industries AG). The solutions (aqueous solution: 20 μL; oil solution: 100 μL) were injected into the channel by air pressure (15 kPa for the aqueous solution; 40 kPa for the oil solution) to generate 25-μm diameter W/O microdroplets for 30 min at 60 Hz. During the operation, microdevices were kept cold with ice. The operation was conducted using custom software, written in Visual Basic. NET 2010 (Microsoft).

### Microscopy

Microdroplet generation was monitored through an objective (UPlanApo 20 ×/0.70 NA, Olympus) by a high-speed camera (LRH2500XE, Digimo) mounted on an inverted microscope (IX-71, Olympus).

Microbial cells were observed using an inverted microscope (IX71, Olympus) with an oil-immersion objective (UPlanApo 40 ×/1.00 NA Oil Iris, Olympus), a xenon lamp and filter sets to observe fluorescence from 4′,6-diamidino-2-phenylindole (DAPI) [excitation filter, FF01-357/44-25 (Semrock); dichroic mirror, FF409-Di03-25 × 36 (Semrock); emission filter, FF02-447/60-25 (Semrock)] and fluorescein [excitation filter, FF01-472/30-25 (Semrock); dichroic mirror, Q505LP (Chroma Technology); emission filter, FF01-520/35-25 (Semrock)]. The bright-field and fluorescence images were captured using an electron multiplying CCD camera (C9100-13, Hamamatsu Photonics). Microbial cell concentrations were estimated by DAPI staining (3 μg/mL, Polysciences) and direct epifluorescence microscopic counting.

### Pick-up of microdroplets and recovery of bacteria exhibiting BGL activity

Microdroplets were plated on a 35-mm glass-based dish (12-mm glass-base, IWAKI) with mineral oil containing 4% (v/v) ABIL EM90 and were observed using a fluorescence microscope. Each microdroplet containing a fluorescent bacterial cell showing BGL activity was picked up and transferred on to the lid of a PCR tube pre-filled with 10 μL of mineral oil using a micromanipulator (CellTram vario, Eppendorf) equipped with an angulated glass capillary (50 μm in inner diameter, Altair Corporation). The cells were recovered from the microdroplets in 4 μL of phosphate-buffered saline by centrifugation (approximately 4,000 rpm) using a bench-top centrifuge (Cubee, Recenttec Inc.).

### Whole genome amplification

Whole genome amplification was performed based on the multiple displacement amplification (MDA) technique^24^,^25^ using the REPLI-g Single Cell Kit (QIAGEN). Briefly, individual cells were lysed with an alkaline solution followed by neutralisation, and the genomic DNAs were amplified using phi29 DNA polymerase at 30 °C for 8 h. No decontamination treatment of amplification reagents and disposables was performed. To confirm the successful amplification of genomes from isolated bacterial cells, 1-μL aliquots of 20-fold diluted MDA products served as templates for PCR of bacterial 16S rRNA genes. PCRs were performed using Tks Gflex DNA Polymerase (Takara Bio) and universal bacterial primers 27F (5′-AGAGTTTGATCMTGGCTCAG-3′) and 1492R (5′-TACGGYTACCTTGTTACGACTT-3′)^26^ as follows: 96 °C for 2 min and 25 cycles of 98 °C for 10 s, 52 °C for 15 s and 68 °C for 90 s. The products were analysed by agarose gel electrophoresis stained with SYBR Safe (Life Technologies). The amplicons were purified (NucleoSpin Gel and PCR Clean-up, Macherey-Nagel GmbH & Co. KG) and subjected to direct sequencing by the Sanger method.

### Genome sequencing and annotation

The resultant MDA products were directly subjected to whole genome sequencing. The sequencing was performed on an Ion Torrent PGM sequencer (Life Technologies) equipped with a 318 chip using 400-base chemistry. The sequence reads were assembled using SPAdes 3.5.0^27^. The genes encoding BGLs were identified with a BLAST search against UniProt (http://www.uniprot.org/) and CAZy (http://www.cazy.org/; ref. 28) databases. Sequences encoding BGLs were confirmed using Sanger sequencing.

### Sequence accession numbers

The 16S rRNA gene sequences obtained in this study were deposited at DDBJ/EMBL/GenBank under the accession numbers LC075346 (SAG_A), LC075347 (SAG_B), LC075348 (SAG_C), LC075349 (SAG_D), LC075350 (SAG_E) and LC075351 (SAG_F). The BGL gene sequences were deposited under the accession numbers LC088483 (BGL1B1), LC088484 (BGL3B1), LC088485 (BGL1C1), LC088486 (BGL3C1), LC088487 (BGL3C2), LC088488 (BGL1D1), LC088489 (BGL3D1) LC088490 (BGL1E1), LC088491 (BGL1E2), LC088492 (BGL3E1), LC088493 (BGL3F1), LC088494 (BGL3F2), LC088495 (BGL3F3) and LC088496 (BGL3F4).

## Results and Discussion

### Method design

**Figure 1:**
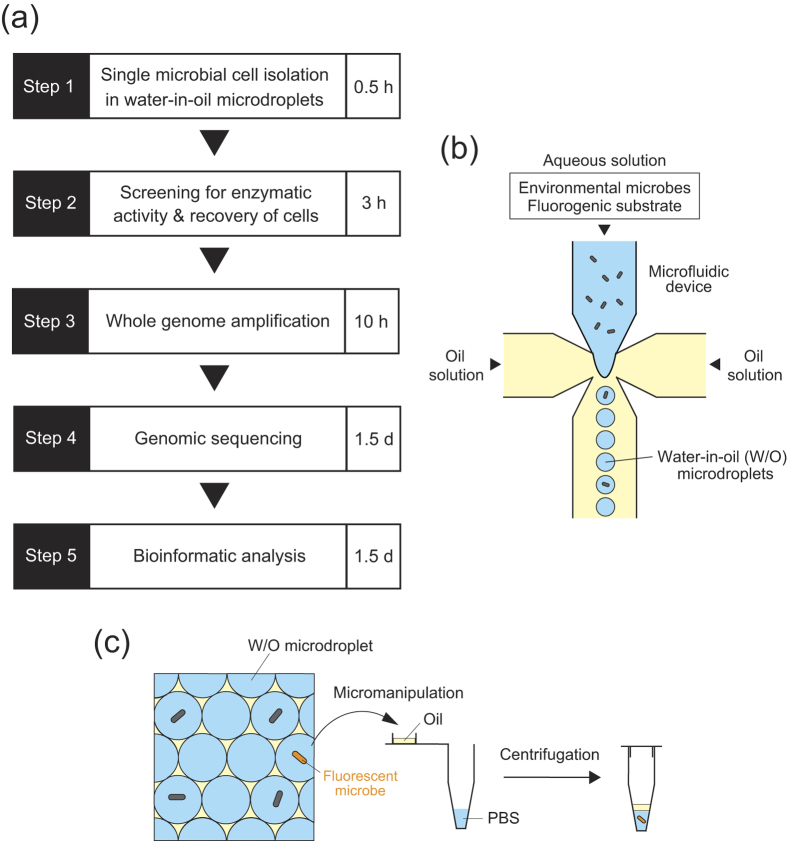
Schematic workflow for identifying microbial enzyme-encoding genes by activity-based single-cell sequencing using microdroplets. (**a**) The workflow includes single microbial cell isolation in W/O microdroplets (step 1), activity-based single-cell screening and recovery of target cells (step 2), whole genome amplification (step 3) and genome sequencing (step 4). Genes encoding the target enzymes are identified based on genomic information (step 5). The entire process could be completed in 4–5 days. (**b**) Schematic representation of single microbial cell isolation in W/O microdroplets using a microfluidic device. (**c**) Schematic representation of activity-based single-cell screening and recovery of target cells.

A schematic representation of our method is shown in Fig. 1a. First, using a microfluidic device, environmental microbes were encapsulated at the single-cell level in picolitre-sized W/O microdroplets, which are aqueous microdroplets dispersed in oil, with a fluorogenic substrate for the target enzyme (Fig. 1a, step 1 and Fig. 1b). Microfluidic systems enabled the production of uniform-sized microdroplets and the rapid isolation of single cells in individual compartments^29^,^30^,^31^. Following incubation at an ambient temperature, the microdroplets were observed under a fluorescence microscope to screen and collect those containing fluorescent microbes that exhibited selected enzymatic activity. This approach enables specific isolation of targeted microbial cells, although they are present at a relatively low abundance in the environment. Each fluorescent microbial cell was recovered from the microdroplets by centrifugation (Fig. 1a, step 2 and Fig. 1c) and then subjected to whole genome amplification using MDA with phi29 DNA polymerase^24^,^25^ (Fig. 1a, step 3). MDA is the preferred method for whole genome amplification of single cells^32^,^33^ and has successfully enabled partial and near-complete genome recovery of microbes from a variety of environments^8^,^9^,^10^,^11^,^12^,^13^. Finally, the resulting MDA products were subjected to high-throughput sequencing (Fig. 1a, step 4), and the sequence data were bioinformatically analysed to identify the genes encoding the target enzymes (Fig. 1a, step 5).

### Identification of novel BGL genes from environmental bacteria

We applied our method to obtain novel BGL genes from bacteria in seawater collected from two different sites: surface seawater and deep seawater. BGLs (EC 3.2.1.21) are found in all domains of living organisms and hydrolyse the β-glycosidic linkages of oligosaccharides, as well as those of alkyl- and aryl β-glucosides. Based on the similarities in their amino acid sequences, BGLs are mainly classified into the glycoside hydrolase family 1 (GH1) and family 3 (GH3) of the CAZy database^28^. BGLs have many potential applications in various biotechnological processes, such as bioethanol production and oligosaccharide synthesis^34^,^35^.

At first, to examine the occurrence rate of BGL-active bacterial cells, the surface seawater was mixed with FDGlu, a fluorogenic substrate for BGL. FDGlu is a membrane-permeable, non-fluorescent molecule. When FDGlu enters bacterial cells expressing BGL, it is hydrolysed to yield fluorescein, which is well retained inside the cells^36^,^37^. Approximately 2% of the cells were considered to be BGL-active, indicating that a large number of cells showed little or no BGL activity (Supplementary Fig. S2).

**Figure 2:**
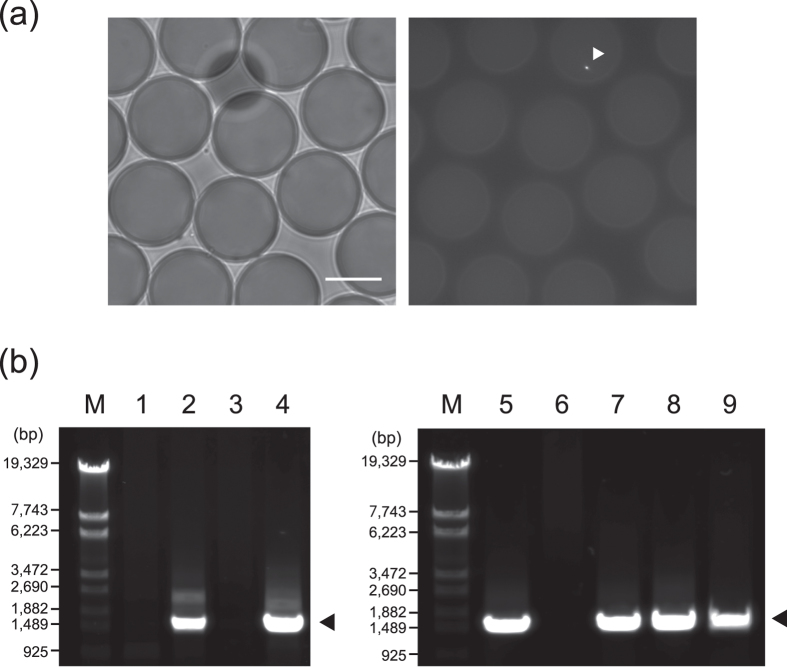
Isolation and genome amplification of bacteria exhibiting BGL activities from surface and deep seawater. (**a**) Bright-field (*left*) and fluorescence (*right*) images of W/O microdroplets encapsulating environmental bacteria with FDGlu. The white arrowhead shows a fluorescent bacterial cell in a W/O microdroplet. Scale bar represents 20 μm. (**b**) PCR amplification of 16S rRNA genes from MDA products. The amplicons were analysed with 1% agarose gel electrophoresis and stained with SYBR Safe. The estimated amplicon size is approximately 1,466 bp. Lane M, DNA marker (λ-*Eco*T14 I digest); lane 1–4, MDA products from surface seawater (Droplet No. 1–4), lane 5–9, MDA products from deep seawater (Droplet No. 5–9).

Next, using microfluidic devices with a flow-focusing junction (Supplementary Fig. S1), bacterial cells in surface and deep seawater were encapsulated with FDGlu in W/O microdroplets (diameter, approximately 25 μm; volume, approximately 8 pL). The cell encapsulation process followed a Poisson distribution as previously reported^38^. To achieve effective cell encapsulation and to avoid the production of a large number of empty microdroplets, bacterial cells in seawater samples were concentrated to approximately 1 × 10^7^ cells/mL by centrifugal ultrafiltration. Ultrafiltration did not have a direct effect on the occurrence rate of fluorescent bacterial cells (Supplementary Fig. S2). In these conditions, bacterial cells were encapsulated at the one-cell-per-ten-microdroplet level, ensuring that few microdroplets contained multiple cells. Of approximately 2 × 10^5^ microdroplets in total with or without bacterial cells, approximately 2 × 10^3^ microdroplets were screened under a fluorescence microscope within 2 h. A total of nine microdroplets containing single fluorescent bacterial cells were picked up using glass capillaries attached to a micromanipulator, and each of the cells were lysed and subjected to MDA. To confirm successful genome amplification from each single bacterial cell isolated from our environmental samples, 16S rRNA gene amplicons derived from each MDA product were sequenced. Two of the four MDA products from surface seawater (Fig. 2b, lanes 2 and 4) and four of the five MDA products from deep seawater (Fig. 2b, lanes 5 and 7–9) produced amplicons. Direct sequencing of the PCR amplicons demonstrated that the genome from each targeted bacterial cell was successfully amplified; three of these were species that had not previously been isolated (<97% homology for 16S rRNA gene sequences on the public database) (Table 1). The PCR-positive MDA products were referred to as single amplified genomes (SAGs): SAG_A, SAG_B, SAG_C, SAG_D, SAG_E and SAG_F. The six SAGs were shotgun sequenced, assembled and analysed. Sequencing and *de novo* assembly results are summarised in Supplementary Table S1. SAG_B–SAG_F contained five genes encoding putative GH1 BGL and eight genes encoding putative GH3 BGL, whereas SAG_A did not contain BGL genes (Table 2). This is probably because certain regions of the genome sequence could not be recovered due to an amplification bias in MDA (Supplementary Table S1). In 12 of the 14 genes obtained, the deduced amino acid sequences were relatively unique, exhibiting 52–74% amino acid sequence identical to putative BGLs found in the public database (Table 2).

We then prepared the recombinant GH1 BGLs (BGL1B1, BGL1C1, BGL1E1 and BGL1E2) to confirm whether the genes identified encode proteins with BGL activity. Their BGL activities were examined with a chromogenic substrate for BGL, *p*-nitrophenyl-β-D-glucopyranoside (*p*NPG), at 30 °C (Supplementary Methods). BGL1C1 and BGL1E2 were significantly less active against pNPG than BGL1B1 and BGL1E1 (Supplementary Table S2). Kinetic parameters for BGL1B1 and BGL1E1 were comparable to or greater than those for GH1 BGLs derived from metagenomes in environments inhabited by BGL-producing bacteria^39^,^40^,^41^,^42^.

### Methodological considerations

We employed a sensitive fluorogenic assay to screen bacterial cells exhibiting BGL activity in microdroplets. We have also demonstrated the successful detection of several enzymatic activities of individual microbial cells using the corresponding fluorogenic substrates in microdroplets (Supplementary Fig. S3). Microdroplets can retain fluorescent products that freely diffuse out of the cell^23^ and allow the screening of secreted enzymes^43^. If a cell-impermeable fluorogenic substrate or a coupled enzyme assay is to be employed, microbial cells have to be lysed by detergent in microdroplets, as previously described^44^. In addition to fluorescence detection, other detection techniques, such as absorbance^45^,^46^, Raman scattering^47^ and electrochemical detection^48^, are compatible with microdroplet-based screening, although they are less sensitive than fluorescence detection. Thus, a wide variety of enzymatic activities can be assayed in microdroplets to identify the genes.

Also, our method has important potential advantages in two fundamental aspects of single-cell genomics: the isolation and genome amplification of single microbial cells. First, our method is capable of the specific isolation of targeted microbial cells. FACS has been the most commonly used high-throughput approach for the separation of individual bacterial cells^9^,^10^,^11^,^12^,^13^. However, environmental samples contain non-cellular fluorescent particles, which can be sorted with targeted microbial cells^14^,^15^. Furthermore, it is difficult to isolate rare microbial cells from environments because conventional FACS systems only collect cells with a fraction of >0.1%^16^,^17^. Although our approach is relatively low-throughput compared with a FACS approach, it permits the visual evaluation of single cells during screening, and rare cells that might be excluded in FACS systems can be recovered. Microdroplets can be collected by micromanipulation using microcapillaries, and the encapsulated microbial cells can be easily recovered from microdroplets by centrifugation using a bench-top centrifuge (Fig. 1c). Second, our method can potentially decrease contamination with non-target microbes and DNA introduced through sample handling (Table 1). In microbial single-cell genomics, one of the most serious problems is contamination; amplification of genomic DNA from a single cell using MDA is susceptible to contamination^26^. The contamination issue has virtually been resolved by the introduction of a highly controlled environment, such as a clean room^9^, the use of liquid-handling robots^9^ and highly-specialized microfluidic platforms^8^,^49^,^50^. In contrast, the risk of contamination can decrease by confining the original sample into W/O microdroplets of a volume of several picolitres. In addition, the oil that surrounds each microdroplet acts as a barrier, preventing crosstalk between cells and contamination from extrinsic sources. From our practical perspective, our method can be performed in a standard biology laboratory with equipment that is commonly available.

We identified BGL genes through a sequence-based approach, which relies on sequence analysis to provide the basis for predictions regarding function. However, it may not identify selected genes that exhibit no sequence similarity to known genes. In this case, an activity-based approach will be beneficial; clones expressing selected activities are screened from libraries constructed from MDA products. This approach is more suitable for obtaining genes with the target enzymatic activities and allows the identification of novel enzymes showing no or little homology to known enzymes, while requiring the expression of the function of interest in heterologous hosts (e.g. *Escherichia coli*) or *in vitro* transcription–translation systems. Moreover, our method can provide more comprehensive information on genetic networks and metabolic pathways of individual microbial cells than conventional metagenomic approaches. Thus, it facilitates the identification of gene clusters encoding metabolically active enzymes.

## Additional Information

**How to cite this article**: Nakamura, K. *et al.* Culture-independent method for identification of microbial enzyme-encoding genes by activity-based single-cell sequencing using a water-in-oil microdroplet platform. *Sci. Rep.* **6**, 22259; doi: 10.1038/srep22259 (2016).

## Supplementary Material

Supplementary Information

## Figures and Tables

**Table 1:** Taxonomic assignment of SAGs based on 16S rRNA sequences.

Droplet No.	SAG	Origin	Accession number	Taxonomy	Sequence identity to the closest relatives*
2	A	Surface seawater	LC075346	*Planctomycetaceae bacterium* D2	96% (AB355061)
4	B	Surface seawater	LC075347	*OM182 bacterium* D4	96% (AY386343)
5	C	Deep seawater	LC075348	*Colwellia* sp. D5	99% (JN175346)
7	D	Deep seawater	LC075349	*Colwellia* sp. D7	99% (JN175346)
8	E	Deep seawater	LC075350	*Colwelliaceae bacterium* D8	96% (HQ203946)
9	F	Deep seawater	LC075351	*Flavobacteriaceae bacterium* D9	98% (EU090719)

The taxonomic assignment of SAGs was performed using SILVA^51^. The bacterium derived from SAG_C (*Colwellia* sp. D5) is closely related to but different from that derived from SAG_D (*Colwellia* sp. D7).

^*^Numbers in parentheses correspond to the GenBank accession numbers.

**Table 2:** Characteristics of deduced BGLs.

	Accession number	Origin	Family	Accession number of the most similar sequences (their origin)	Identity (%)
BGL1B1	LC088483	SAG_B	GH1	WP_015935647* (*Arthrobacter chlorophenolicus*)	56
BGL3B1	LC088484	SAG_B	GH3	WP_028040971 (*Caulobacter* sp. URHA0033)	52
BGL1C1	LC088485	SAG_C	GH1	WP_011044459 (*Colwellia psychrerythraea*)	74
BGL3C1	LC088486	SAG_C	GH3	WP_010381006 (*Pseudoalteromonas rubra*)	63
BGL3C2	LC088487	SAG_C	GH3	WP_011044492 (*Colwellia psychrerythraea*)	67
BGL1D1	LC088488	SAG_D	GH1	WP_033093338 (*Colwellia psychrerythraea*)	74
BGL3D1	LC088489	SAG_D	GH3	WP_011044492 (*Colwellia psychrerythraea*)	68
BGL1E1	LC088490	SAG_E	GH1	WP_019026132 (*Colwellia piezophila*)	70
BGL1E2	LC088491	SAG_E	GH1	WP_010557357 (*Pseudoalteromonas marina*)	72
BGL3E1	LC088492	SAG_E	GH3	KGJ93128 (*Colwellia psychrerythraea*)	68
BGL3F1	LC088493	SAG_F	GH3	KGL60449 (*Polaribacter* sp. Hel1_33_49)	95
BGL3F2	LC088494	SAG_F	GH3	WP_036785255 (*Polaribacter* sp. Hel1_33_49)	99
BGL3F3	LC088495	SAG_F	GH3	KGE87595 (*Phaeodactylibacter xiamenensis*)	63
BGL3F4	LC088496	SAG_F	GH3	WP_036784331 (*Polaribacter* sp. Hel1_33_49)	73

Sequence homology searches were performed using the program Protein BLAST (BLASTP). *Functionally characterised.
