# Supplementary material for: Culture-independent method for identification of microbial enzyme-encoding genes by activity-based single-cell sequencing using a water-in-oil microdroplet platform
Source: Sci Rep. 2016 Feb 26;6:22259. doi: 10.1038/srep22259 (PMC4768102; doi:10.1038/srep22259)
Supplement: Supplementary Information [file srep22259-s1.pdf]

Supplementary information

Culture-independent method for identification of microbial enzyme-encoding genes by activity-based single-cell sequencing using a water-in-oil microdroplet platform

Kazuki Nakamura[1], Ryo Iizuka[1*], Shinro Nishi[2], Takao Yoshida[2], Yuji Hatada[2], Yoshihiro Takaki[2], Ayaka Iguchi[3], Dong Hyun Yoon[3], Tetsushi Sekiguchi[4], Shuichi Shoji[3] and Takashi Funatsu[1*]

[1]Graduate School of Pharmaceutical Sciences, The University of Tokyo, 7-3-1, Hongo, Bunkyo-ku, Tokyo 113-0033, Japan; [2]Japan Agency for Marine-Earth Science and Technology, 2-15 Natsushima-cho, Yokosuka-shi, Kanagawa 237-0061, Japan; [3]Department of Nanoscience and Nanoengineering (ASE Graduate School), Waseda University, 3-4-1 Okubo, Shinju-ku, Tokyo 169-8555, Japan; [4]Research Organization for Nano & Life Innovation, Waseda University, 513, Waseda-tsurumaki-cho, Shinjuku-ku, Tokyo, 162-0041, Japan

*Address correspondence to: Ryo Iizuka, Graduate School of Pharmaceutical Sciences, The University of Tokyo, 7-3-1 Hongo, Bunkyo-ku, Tokyo 113-0033, Japan, TEL: +81-3-5841-4761, FAX: +81-3-5802-3339, E-mail: iizuka@mol.f.u-tokyo.ac.jp; Takashi Funatsu, Graduate School of Pharmaceutical Sciences, The University of Tokyo, 7-3-1 Hongo, Bunkyo-ku, Tokyo 113-0033, Japan, TEL: +81-3-5841-4760, FAX: +81-3-5802-3339, E-mail: funatsu@mol.f.u-tokyo.ac.jp

Supplementary Methods

Construction of expression plasmids for BGLs

The coding sequences of deduced GH1 BGLs (BGL1B1, BGL1C1, BGL1E1 and BGL1E2) were amplified from MDA products using appropriate primer sets to introduce an *Nde* I restriction site at the 5′-end and a *Bam*H I or *Eco*R I restriction site at the 3′-end (Supplementary Table S3). The amplified fragment was digested with *Nde* I and *Bam*H I or *Eco*R I and was ligated to the same sites in the pET21c vector (Novagen). The expression plasmids for BGLs with a hexahistidine-tag at the C-terminus were obtained using the KOD –Plus– Mutagenesis Kit (Toyobo) using the expression plasmids for BGLs as a template. The oligonucleotides used for incorporating a hexahistidine-tag are listed in Supplementary Table S4.

Expression and purification of BGLs

E. coli BL21-CodonPlus (DE3) cells (Toyobo) carrying the expression plasmids were grown in Luria–Bertani medium containing 100 μg/mL ampicillin at 37°C until the OD_{600} reached 0.5–0.6 and were then cultivated in the presence of 0.5 mM isopropylthio β-D-1-galactoside (IPTG) for 18 h at 15°C to express BGLs with a hexahistidine-tag in the soluble fraction. The harvested cells were suspended in buffer A [25 mM HEPES-NaOH (pH 7.5), 1 mM $MgCl_2$ and 500 mM NaCl] containing 1 mM benzamidine (Nacalai Tesque) and 200 μM 4-(2-aminoethyl) benzenesulfonyl fluoride hydrochloride (Nacalai Tesque) and were disrupted by sonication on ice. The supernatant after centrifugation (120,000 g, 60 min, 4°C) was applied to a HisTrap chelating HP column (GE Healthcare UK Ltd.) equilibrated with buffer A. Proteins were eluted using a linear gradient of 20–500 mM imidazole in the same buffer. The concentrated fractions were loaded on to a gel filtration column (HiLoad 26/60 Superdex 200 prep grade, GE Healthcare UK Ltd.) equilibrated with buffer B [20 mM HEPES-NaOH (pH 7.5), 150 mM NaCl and 1 mM DTT]. Purified BGLs were concentrated by ultrafiltration and stored in 20% (v/v) glycerol at −80°C before use. Their concentrations were determined from the absorbance at 280 nm with sequence-deduced molar extinction coefficients (calculated using Protein Calculator; http://protcalc.sourceforge.net/).

Measurement of BGL activity

The enzymatic activity of purified BGLs was measured using *p*NPG (Sigma-Aldrich) as a substrate at 30°C in 50 mM MES-NaOH (pH 6.5) and 50 mM NaCl. The enzymatic reactions were initiated by adding BGLs. The reactions were terminated by mixing with 0.2 M Na_2CO_3, and the absorbance at 400 nm was then measured using a spectrophotometer (V-670,

JASCO). For blank experiments, the same procedures were performed without BGLs. The amount of released *p*-nitrophenol was calculated by subtracting the blank value and using a molar extinction coefficient of 17,100 $M^{-1} \cdot cm^{-1}$. Data were fitted to the Michaelis–Menten equation using the KaleidaGraph program (Synergy Software) to determine kinetic parameters. Experiments were performed in triplicate.

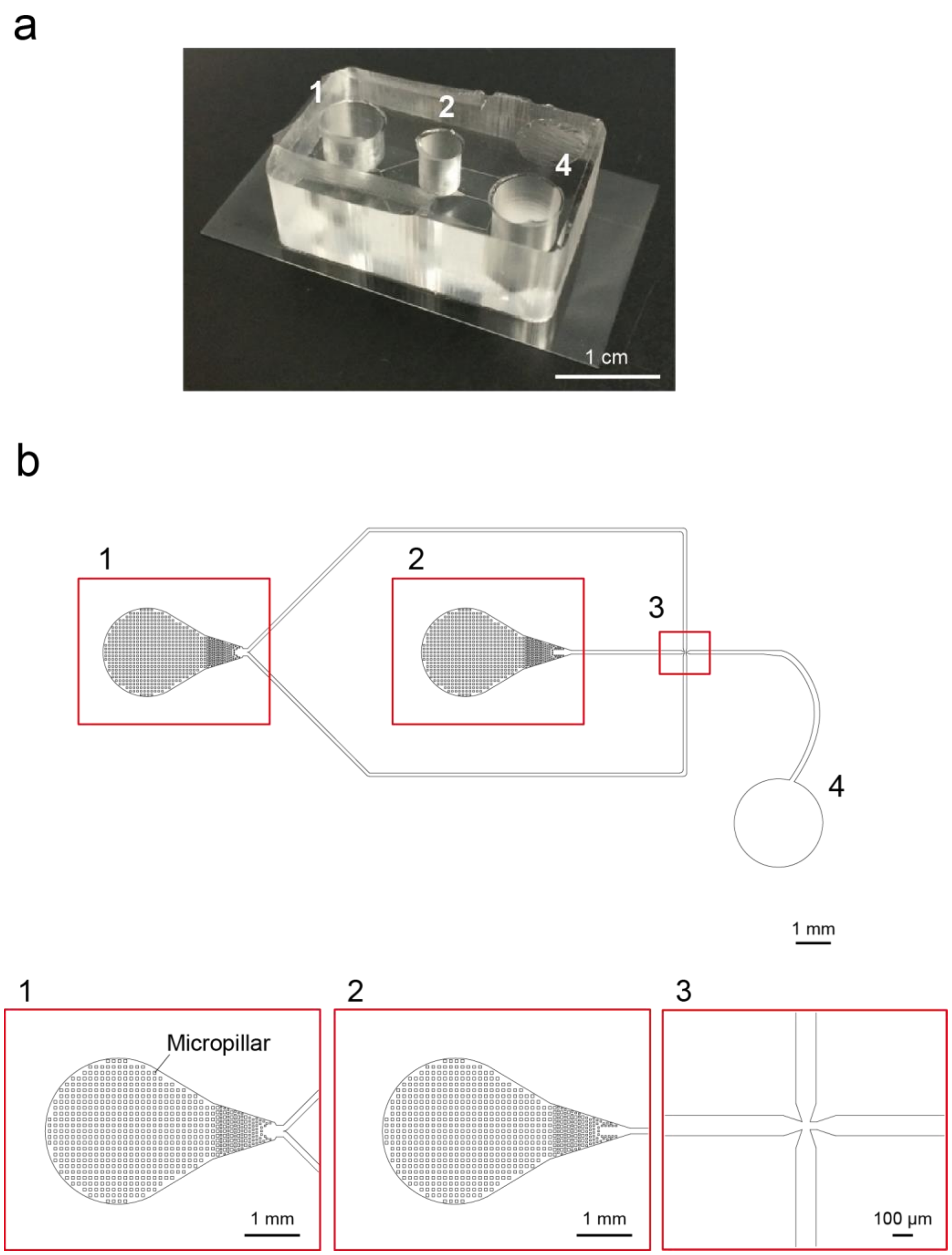

Supplementary Fig. S1. Microfluidic device used in this study

(**a**) Photograph of the microfluidic device. (**b**) Design of the microfluidic device. 1, Oil inlet; 2, aqueous inlet; 3, flow-focusing junction; and 4, device outlet. The aqueous phase is continuously sheared off at a flow-focusing junction (3) by the oil stream to generate W/O microdroplets. At the device outlet (4), microdroplets can be collected using a micropipette. To prevent clogging of the junction, passive filters consisting of micropillars are incorporated upstream of the channels (1, 2). The width of the main channels was 100 µm, and the width at the flow-focusing constrictions was 40 µm. The height of all channels was 50 µm.

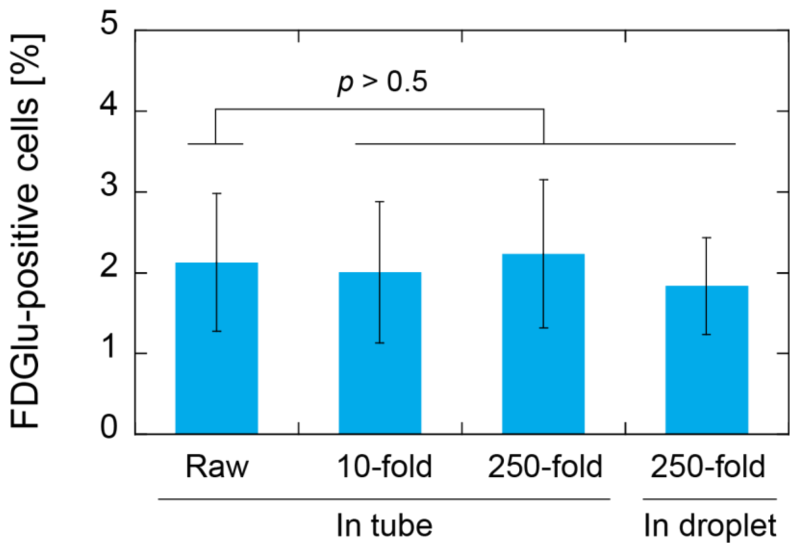

Supplementary Fig. S2. Percentage of FDGlu-positive cells in concentrated seawater

Surface seawater was collected from the coast of Tokyo Bay, Japan (35° 19.170′ N, 139° 39.068′ E) in November 2015. The surface seawater was passed through a 41-μm nylon net filter, a 20-μm nylon net filter and a 10-μm Omnipore membrane filter to separate large particles and debris. The aliquot (approximately 150 mL) was ultrafiltrated (5,000 *g*, 1–2 h, 4˚C) using a 10-kDa pore membrane (Amicon Ultra-15). The seawater samples were mixed with DAPI (1.7 μg/mL) and FDGlu (1.9 mM) in PCR tubes or W/O microdroplets (diameter: approximately 35 μm). The stained cells were observed under a fluorescence microscope to calculate the percentage of FDGlu-positive cells versus the total number of cells stained by DAPI. Raw denotes raw seawater sample (approximately 1.3×10^6 cells/mL), 10-fold denotes 10-fold concentrated seawater (approximately 2.0×10^7 cells/mL) and 250-fold denotes 250-fold concentrated seawater (approximately 3.4×10^8 cells/mL). Results are shown as mean ± standard deviation of three independent experiments. For each individual experiment, at least 240 cells were analysed. There was no statistically significant difference among them ($p > 0.5$, Student's *t*-test).

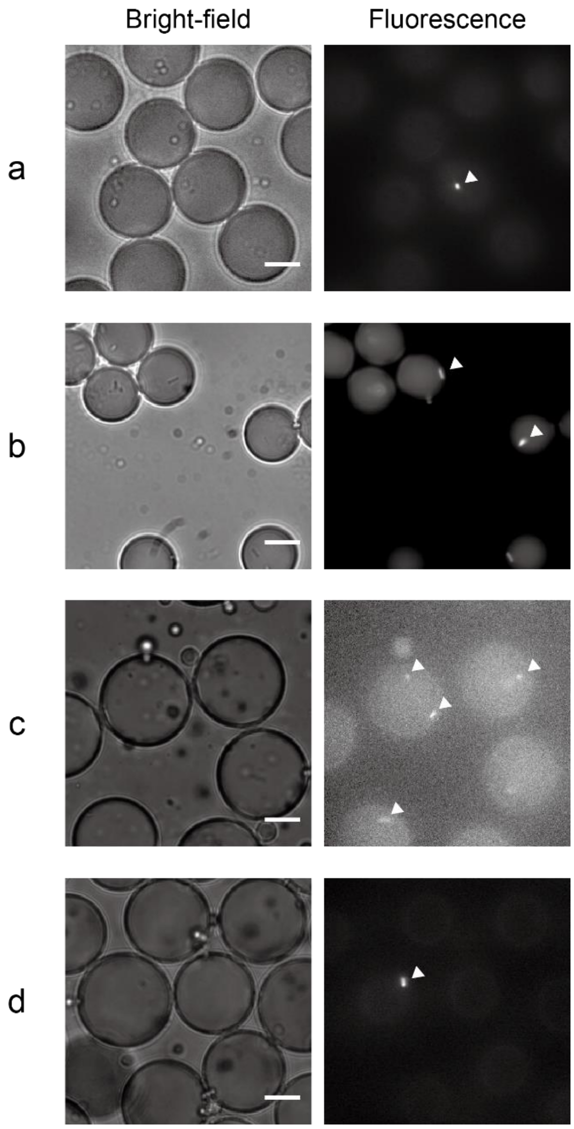

Supplementary Fig. S3. Detection of enzymatic activities at the single-cell level in W/O microdroplets

(**a**) Detection of esterase activity using 5(6)-carboxyfluorescein diacetate (Dojindo Laboratories). *E. coli* XL10-Gold were encapsulated into W/O microdroplets with the fluorogenic substrate. (**b**) Detection of phosphatase activity using fluorescein diphosphate (Marker Gene Technologies). *E. coli* XL10-Gold were encapsulated into W/O microdroplets with the fluorogenic substrate. (**c**) Detection of protease activity using Rhodamine 110, bis-(N-CBZ-L-arginine amide) (Biotium). *E. coli* XL10-Gold were encapsulated into W/O microdroplets with the fluorogenic substrate. (**d**) Detection of β-galactosidase activity using fluorescein di-β-D-galactopyranoside (Marker Gene Technologies). *E. coli* BL21(DE3) were encapsulated into W/O microdroplets with the fluorogenic substrate and IPTG. White arrowheads show fluorescent *E. coli* cells in W/O microdroplets. Scale bars represent 10 μm.

Supplementary Table S1. Sequencing and assembly results of the SAGs

	SAG_A	SAG_B	SAG_C	SAG_D	SAG_E	SAG_F
**Sequencing results**						
**Total reads**	1,137,441	638,895	766,115	719,809	623,599	782,795
**Average read length (bp)**	287.2	306.3	288.2	290.7	332.2	301.2
**Total read bases (bp)**	326,656,694	195,670,070	220,759,118	209,221,118	207,177,099	235,759,040
**Assembly results**						
**Assembled contigs**	1,929	4,730	997	4,264	8,342	243
**Contigs (>500 bp)**	527	969	528	1,417	2,058	234
**N50 (bp)***	6,862	6,647	19,912	4,174	2,175	45,833
**Maximum length (bp)**	50,754	60,840	73,150	28,157	32,637	103,055
**Total bases (bp)**	2,286,206	4,369,119	4,341,705	4,543,524	5,689,244	3,166,946
**Average read depth**	156.4	29.9	24.0	32.8	26.0	29.4
**GC content (%)**	47.3	50.5	36.2	36.2	35.3	32.8

The section of total bases in the assembly results represents the number of bases that comprised the draft genome. *N50 represents the length of contigs that collectively cover at least 50% of the assembly.

Supplementary Table S2. Kinetic parameters for the enzymatic hydrolysis of *p*NPG

	K_m (mM)	k_{cat} (1/s)	k_{cat}/K_m (1/M·s)
BGL1B1	2.23 ± 0.387	13.3 ± 1.65	5,950 ± 1,270
BGL1C1	1.61 ± 0.152	0.0145 ± 0.000435	9.05 ± 0.899
BGL1E1	1.04 ± 0.104	28.4 ± 1.97	26,800 ± 3,220
BGL1E2	1.24 ± 0.124	0.0217 ± 0.000939	17.5 ± 1.91

Data show the mean and standard deviation from three independent experiments.

Supplementary Table S3. Primers for cloning GH1 BGL genes

Primer set		Sequence (5′–3′)	Enzyme
**BGL1B1**	Forward	GGAATTCCATATGGAATCGTACTTGTTTCCCG	*Nde* I
	Reverse	GGAATTCTCAGCGCAGGGTGTTG	*Eco*R I
**BGL1C1**	Forward	GGAATTCCATATGCTAAAAAAAGAATTCATCTATGGC	*Nde* I
	Reverse	CGGGATCCTTAGCGATTACTGATGAGCGTTTTATAC	*Bam*H I
**BGL1E1**	Forward	GGAATTCCATATGAAAATAGAATTACCTAAAGAATCGAC	*Nde* I
	Reverse	GGAATTCTTAAGGAGTTATTAGGCGTTGTTTAATAAAATC	*Eco*R I
**BGL1E2**	Forward	GGAATTCCATATGAATACGTTTGCATTACCACTG	*Nde* I
	Reverse	CGGGATCCTTAACGCAAAACCATACCCCTC	*Bam*H I

Restriction enzyme recognition sites are underlined.

Supplementary Table S4. Primers for incorporating a hexahistidine-tag

Primer set		Sequence (5′–3′)
**BGL1B1**	Forward	GCGCAGGGTGTTGCTGCGGG
	Reverse	CACCATCACCATCACCATTGAGAATTCGAGCTCCGTCGACAAGCT
**BGL1C1**	Forward	GCGATTACTGATGAGCGTTTTATACGCATGACCAC
	Reverse	CACCATCACCATCACCATTAAGGATCCGAATTCGAGCTCCGTCGACA
**BGL1E1**	Forward	AGGAGTTATTAGGCGTTGTTTAATAAAATCACTATATAACAGTCCACTGTG
	Reverse	CACCATCACCATCACCATTGAGAATTCGAGCTCCGTCGACAAGCT
**BGL1E2**	Forward	ACGCAAAACCATACCCCTCATGTCCTTTTTC
	Reverse	CACCATCACCATCACCATTAAGGATCCGAATTCGAGCTCCGTCGACA

The underlined sequences encode a hexahistidine-tag.
